# Hypertension in Florida: Data From the OneFlorida Clinical Data Research Network

**DOI:** 10.5888/pcd15.170332

**Published:** 2018-03-01

**Authors:** Steven M. Smith, Kathryn McAuliffe, Jaclyn M. Hall, Caitrin W. McDonough, Matthew J. Gurka, Temple O. Robinson, Ralph L. Sacco, Carl Pepine, Elizabeth Shenkman, Rhonda M. Cooper-DeHoff

**Affiliations:** 1 Department of Pharmacotherapy and Translational Research, College of Pharmacy, University of Florida, Gainesville, Florida; 2 Department of Community Health and Family Medicine, College of Medicine, University of Florida, Gainesville, Florida; 3 Department of Health Outcomes and Biomedical Informatics, College of Medicine, University of Florida, Gainesville, Florida; 4 Bond Community Health Center, Inc, and College of Medicine, Florida State University, Tallahassee, Florida; 5 Departments of Neurology and Public Health Sciences, Miller School of Medicine, University of Miami, Miami, Florida; 6 Division of Cardiovascular Medicine, Department of Medicine, College of Medicine, University of Florida, Gainesville, Florida

## Abstract

**Introduction:**

Hypertension is highly prevalent in Florida, but surveillance through the Behavioral Risk Factor Surveillance System (BRFSS) is limited to self-reported hypertension and does not capture data on undiagnosed hypertension or measure blood pressure. We aimed to characterize the hypertensive population in the OneFlorida Clinical Research Consortium by using electronic health records and provide proof-of-concept for using routinely collected clinical data to augment surveillance efforts.

**Methods:**

We identified patients with hypertension, defined as having at least 1 outpatient visit from January 2012 through June 2016 with an ICD-9-CM or ICD-10-CM diagnosis code for hypertension, or in the absence of a diagnosis, an elevated blood pressure (systolic ≥140 mm Hg or diastolic ≥90 mm Hg) recorded in the electronic health record at the most recent visit. The hypertensive population was characterized and mapped by zip code of patient residence to county prevalence.

**Results:**

Of 838,469 patients (27.9% prevalence) who met the criteria for hypertension, 68% had received a diagnosis and 61% had elevated blood pressure. The geographic distribution of hypertension differed between diagnosed hypertension (highest prevalence in northern Florida) and undiagnosed hypertension (highest prevalence along eastern coast, in southern Florida, and in some rural western Panhandle counties). Uncontrolled hypertension was concentrated in southern Florida and the western Panhandle.

**Conclusion:**

Our use of clinical data, representing usual care for Floridians, allows for identifying cases of uncontrolled hypertension and potentially undiagnosed cases, which are not captured by existing surveillance methods. Large-scale pragmatic research networks, like OneFlorida, may be increasingly important for tailoring future health care services, trials, and public health programs.

## Introduction

Hypertension is a leading modifiable risk factor for cardiovascular disease, chronic kidney disease, stroke, and death (1,2) and the most common condition in Florida, affecting nearly half (48.7%) of adults aged 45 to 79 (3). Reducing blood pressure mitigates the substantial adverse sequelae of hypertension, yet nationwide, 16% of adults with hypertension are unaware that they have it, 24% are untreated, and only 54% achieve blood pressure control of 140/90 mm Hg (4). The risk of hypertension-associated sequelae is particularly high in Florida, a state that ranks among the worst states in hypertension prevalence (3). Reducing Florida’s hypertension prevalence to a level similar to that of the best states (approximately 41% for men and 36% or women) could prevent up to 10% of all cardiovascular-related deaths in Florida (3). Achieving greater blood pressure control is considered a high priority statewide (5).

The implementation of public health programs to mitigate the burden of hypertension is hampered by a lack of information about the disease’s regional distribution, especially the distribution of undiagnosed and uncontrolled hypertension. Most published data on these measures come from surveys conducted nationally, which, because of their sampling design, do not allow for detailed analysis below the state level (ie, National Health and Nutrition Examination Survey [NHANES]) or provide data on self-reported measures of hypertension but no clinical correlates (ie, Behavioral Risk Factor Surveillance System [BRFSS]). Thus, a gap exists for regional or statewide evidence, and new data sources are needed to better surveil disease prevalence, treatment, and health inequities at the state and local level (6).

The development of large-scale clinical research networks has created opportunities for studying diseases and their treatments through use of “real world” electronic health record (EHR) data. Such networks, particularly those that integrate clinical data from routine medical care, can augment existing surveillance efforts with more detailed, longitudinal patient-level data. We examined data on hypertension in the OneFlorida Clinical Research Consortium (hereinafter, referred to as OneFlorida), a collaborative research network of health systems, providers, and insurers in Florida, the third most populous state, which has a diverse population and is a bellwether for demographic trends. The primary objective of this study was to characterize the hypertensive population in OneFlorida (7). Second, we aimed to provide proof-of-concept for use of clinical data research networks in the surveillance of common chronic diseases like hypertension.

## Methods

We performed a retrospective cross-sectional study of data on adult patients in the OneFlorida Data Trust, a component of OneFlorida (7). OneFlorida is one of 13 clinical data research networks in the United States funded by the Patient-Centered Outcomes Research Institute that constitute the National Patient-Centered Clinical Research Network (PCORnet) (8). OneFlorida is a partnership of 11 health systems and affiliated practices in Florida, several statewide insurance programs, and Florida’s Agency for Health Care Administration, which oversees Florida Medicaid (9). These partners provide care for more than 10 million Floridians, nearly half of all Florida residents. OneFlorida partners contribute clinical or administrative claims data quarterly to the OneFlorida Data Trust (10), a secure, centralized data repository maintained at the University of Florida that integrates OneFlorida’s EHR data into PCORnet’s common data model. This study was approved by OneFlorida’s institutional review board at the University of Florida.

### Cohort development

We included adults aged 18 or older with at least 1 ambulatory visit or other outpatient encounter from January 1, 2012, through June 30, 2016. This period encompasses the earliest date for which all partners provided data to the Data Trust (January 2012) through the most recent date (June 2016) for which the Data Trust data had been certified as research ready by the PCORnet Coordinating Center at Duke University. To be certified as research ready, data must meet predetermined thresholds for the percentage of fields containing valid data, the patterns of health care use among patients, and the amount of missing data. We included only patient visits coded as ambulatory visits or outpatient encounters. Being designated as hypertensive was operationalized by 1) an ICD-9-CM *(International Classification of Diseases, Ninth Revision, Clinical Modification* [11]) (401, 401.0, 401.1, or 401.9) or ICD-10-CM (*International Classification of Diseases, Tenth Revision, Clinical Modification* [12]) (I10) diagnosis code or 2) a systolic blood pressure measurement of 140 mm Hg or more or diastolic blood pressure of 90 mm Hg or more in the EHR during the most recent ambulatory visit or outpatient encounter. Patient-reported blood pressure measurements were not considered. We did not include use of antihypertensive medication as a criterion because these data were not available from all OneFlorida partners. Systolic blood pressure values of less than 70 mm Hg or greater than 250 mm Hg were considered erroneous, recoded as missing, and excluded, as were diastolic blood pressure values of less than 50 mm Hg or more than 150 mm Hg.

We extracted the following data from the EHR of the most recent visit in which a patient met our criteria for hypertension: demographic characteristics (age, race, ethnicity, sex, zip code of residence), recorded blood pressure, and clinical characteristics (body mass index [BMI] and comorbidities). If BMI was not recorded, we calculated BMI from weight and height measurements at that visit. The following measurements were considered errors, recoded as missing, and excluded from analysis: BMI of less than 5 or 90 or more, a height of less than 3 feet or 8 feet or more, and a weight of 40 pounds or less or 500 pounds or more. We also assessed the type of antihypertensive medications used by each patient during the 4.5-year study period.

### Analysis

We used descriptive statistics to characterize the OneFlorida hypertensive population overall and by age group (18–44 years, 45–64, 65–74, and ≥75). Hypertension prevalence was defined as the proportion of patients meeting our hypertension criteria among all patients with at least 1 ambulatory visit or outpatient encounter during the study period. Prevalence of uncontrolled blood pressure was defined as the proportion of patients with systolic blood pressure of 140 mm Hg or more or diastolic blood pressure of 90 mm Hg or more, or both, among all patients with hypertension who had at least 1 valid blood pressure measurement recorded. No weightings were applied for the overall characterization. Because our intention was to assess the suitability of using OneFlorida data to augment existing surveillance efforts by qualitatively assessing the extent to which these data accord with data from previous research, we opted not to conduct statistical tests for comparisons across age groups or other subgroups.

Using zip code of patient’s residence, we aggregated counts (hypertension prevalence, uncontrolled blood pressure prevalence) to Zip Code Tabulation Areas (ZCTAs) and county equivalents. Of 983 ZCTAs in Florida that contain residential housing, 90% exist completely within the boundary of a single county. ZCTAs that are bisected by a county boundary comprise only 1% of Florida’s population. We matched zip codes to counties by using the US Census Bureau relationship files and constructed county-level equivalent counts of hypertension prevalence and uncontrolled blood pressure prevalence. ZCTA counts were aggregated to create county rates, by quintile, and mapped, by using ArcGIS (Esri) to characterize distributions geographically.

## Results

**Figure 1:**
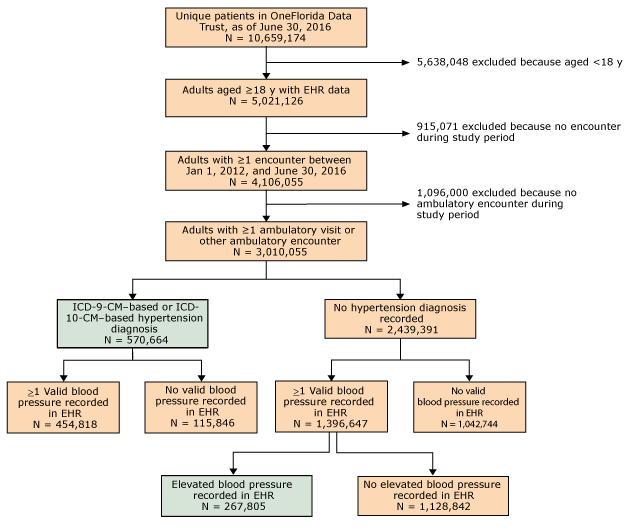
Flow diagram for OneFlorida hypertensive cohort identification. Abbreviations: EHR, electronic health record; ICD-9-CM, *International Classification of Diseases, Ninth Revision, Clinical Modification* (11); ICD-10-CM, *International Classification of Diseases, Tenth Revision, Clinical Modification* (12).

Among 3.01 million adults included in this analysis, 838,469 were characterized as having hypertension. Of these, 570,664 (68.1%) were based on ICD-9-CM or ICD-10-CM diagnosis and 267,805 (31.9%) were based on blood pressure measurements (n = 159,441 [19.0%] based on systolic blood pressure ≥140 mm Hg; n = 39,133 [4.7%] based on a diastolic blood pressure ≥90 mm Hg; and n = 69,231 [8.3%] based on systolic blood pressure ≥140 mm Hg and diastolic blood pressure ≥90 mm Hg) (Figure 1). The overall prevalence of hypertension was 27.9%, and ranged from 11.1% among those aged 18 to 44 to 48.1% among those aged 75 or older (Table 1).

**Table 1:** Characteristics of Adults With Hypertension in the OneFlorida Data Trust, January 2012–June 2016^a^

Characteristic	Age Group, y				Total
	18–44	45–64	65–74	≥75	
**Total eligible population^b^**	1,190,542	1,046,467	421,897	351,149	3,010,055
**Met criteria for hypertension**	132,459 (11.1)	350,448 (33.5)	186,737 (44.3)	168,825 (48.1)	838,469 (27.9)
≥1 ICD-9-CM or ICD-10-CM diagnosis^c^	66,807 (50.5)	245,451 (70.0)	135,213 (72.4)	123,193 (73.0)	570,664 (68.1)
Systolic blood pressure ≥140 mm Hg^c^^,^ d	25,968 (19.6)	54,530 (15.6)	39,407 (21.1)	39,536 (23.4)	159,441 (19.0)
Diastolic blood pressure ≥90 mm Hg^c^^,^ d	19,030 (14.4)	16,623 (4.7)	2,422 (1.3)	1,058 (0.6)	39,133 (4.7)
Systolic blood pressure ≥140 and diastolic blood pressure ≥90 mm Hg^c^^,^ d	20,654 (15.6)	33,844 (9.7)	9,695 (5.2)	5,038 (3.0)	69,231 (8.3)
**Demographic^c^**					
Female	68,300 (51.6)	185,605 (53.0)	99,132 (53.1)	97,388 (57.7)	450,425 (53.7)
Race					
White	74,477 (56.2)	219,302 (62.6)	132,254 (70.8)	123,793 (73.3)	549,826 (65.6)
Black	40,376 (30.5)	86,307 (24.6)	29,704 (15.9)	19,810 (11.7)	176,197 (21.0)
Other	906 (0.7)	2,498 (0.7)	1,028 (0.6)	595 (0.4)	5,027 (0.6)
Unknown	16,700 (12.6)	42,341 (12.1)	23,751 (12.7)	24,627 (14.6)	107,419 (12.8)
Ethnicity					
Hispanic	26,252 (19.8)	70,011 (20.0)	29,293 (15.7)	24,475 (14.5)	150,031 (17.9)
Non-Hispanic	98,707 (74.5)	263,062 (75.1)	147,831 (79.2)	133,561 (79.1)	643,161 (76.7)
Unknown	7,498 (5.7)	17,373 (5.0)	9,612 (5.1)	10,787 (6.4)	45,270 (5.4)
**Blood pressure^e^**	120,400 (90.9)	305,500 (87.2)	159,661 (85.5)	137,062 (81.2)	722,623 (86.2)
Systolic blood pressure, mm Hg, mean (SD)	138 (16)	138 (18)	140 (20)	141 (21)	139 (19)
Diastolic blood pressure, mm Hg, mean (SD)	86 (11)	82 (11)	77 (11)	73 (11)	80 (12)
Systolic blood pressure ≥140 and diastolic blood pressure ≥90 mm Hg^f^	31,455 (26.1)	62,984 (20.6)	17,521 (11.0)	9,077 (6.6)	121,037 (16.7)
Systolic blood pressure ≥140 or diastolic blood pressure ≥90 mm Hg^f^	55,534 (46.1)	117,709 (38.5)	74,340 (46.6)	72,875 (53.2)	320,458 (44.3)
**Body mass index,^e^ kg/m^2^**	122,338 (92.4)	319,259 (91.1)	167,776 (89.8)	146,561 (86.8)	755,934 (90.2)
<25.0^f^	19,581 (16.0)	49,276 (15.4)	33,847 (20.2)	50,764 (34.6)	153,468 (20.3)
25.0 to <30.0^f^	30,162 (24.7)	94,186 (29.5)	57,568 (34.3)	54,951 (37.5)	236,867 (31.3)
≥30.0^f^	72,595 (59.3)	175,797 (55.1)	76,361 (45.5)	40,846 (27.9)	365,599 (48.4)
**Comorbidities^c^**					
Diabetes	15,313 (11.6)	85,155 (24.3)	55,596 (29.8)	47,149 (27.9)	203,213 (24.2)
Coronary artery disease	2,267 (1.7)	33,224 (9.5)	33,610 (18.0)	42,662 (25.3)	111,763 (13.3)
Chronic kidney disease	4,626 (3.5)	20,406 (5.8)	17,791 (9.5)	26,021 (15.4)	68,844 (8.2)
Dyslipidemia	21,483 (16.2)	130,130 (37.1)	89,600 (48.0)	84,609 (50.1)	325,822 (38.9)
Peripheral arterial disease	1,014 (0.8)	10,776 (3.1)	10,797 (5.8)	13,517 (8.0)	36,104 (4.3)
Angina	1,417 (1.1)	12,780 (3.7)	8,738 (4.7)	7,735 (4.6)	30,670 (3.7)
Myocardial infarction	1,124 (0.9)	12,052 (3.4)	9,695 (5.2)	11,285 (6.7)	34,156 (4.1)
Stroke	1,239 (0.9)	8,199 (2.3)	6,057 (3.2)	8,383 (5.0)	23,878 (2.9)
Major depression	7,478 (5.7)	22,712 (6.5)	8,323 (4.5)	5,551 (3.3)	44,064 (5.3)
Minor depression	10,469 (7.9)	35,294 (10.1)	16,059 (8.6)	13,425 (8.0)	75,247 (9.0)
Dementia	71 (0.1)	1,213 (0.4)	3,123 (1.7)	15,886 (9.4)	20,293 (2.4)
Memory loss	701 (0.5)	3,511 (1.0)	3,165 (1.7)	6,099 (3.6)	13,476 (1.6)
Chronic heart failure	2,031 (1.5)	13,794 (3.9)	11,706 (6.3)	21,217 (12.6)	48,748 (5.8)

Abbreviations: ICD-9-CM, *International Classification of Diseases, Ninth Revision, Clinical Modification* (11); ICD-10-CM, *International Classification of Diseases, Tenth Revision, Clinical Modification* (12); SD, standard deviation.

a Values are number (percentage) unless otherwise indicated.

b All patients in OneFlorida with at least 1 ambulatory care visit at which blood pressure was measured.

c Percentage in each cell was calculated as the cell number (numerator) divided by the corresponding cell number for “Met criteria for hypertension” (denominator).

d Category excludes patients with a hypertension diagnosis (ICD-9-CM or ICD-10-CM).

e Row describes number (% of all patients with hypertension) with nonmissing data for this variable.

f Percentage in each cell was calculated as the cell number (numerator) divided by the corresponding number of patients with nonmissing data (denominator) for the variable.

**Figure 2:**
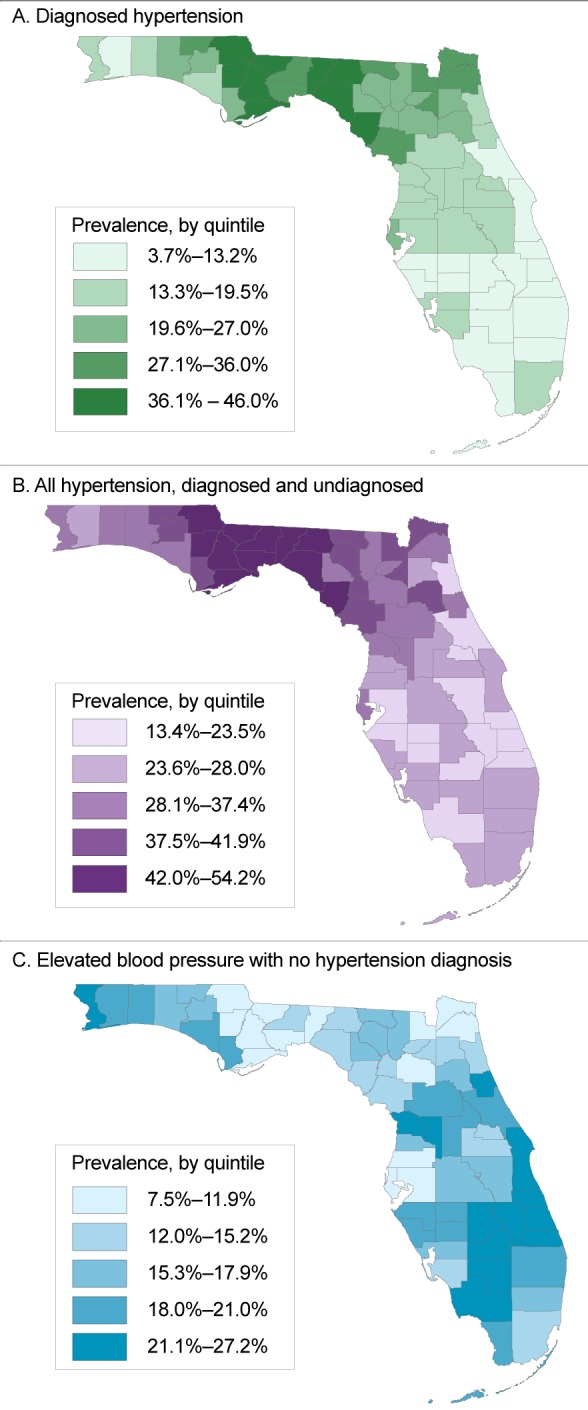
Prevalence, by quintile, of hypertension among patients with at least 1 ambulatory visit or outpatient encounter recorded from January 1, 2012, through June 30, 2016, in OneFlorida, a partnership of 11 health systems and affiliated practices in Florida, by county. Panel A, prevalence of diagnosed hypertension; panel B, prevalence of hypertension, both diagnosed and undiagnosed; panel C, prevalence of elevated blood pressure but no hypertension diagnosis. **Table:** 

Counties with the highest prevalence of diagnosed hypertension and the highest overall prevalence of hypertension (diagnosed and undiagnosed) were concentrated primarily in northern Florida (Figure 2, panel A and panel B). In contrast, the highest prevalence of elevated blood pressure with no hypertension diagnosis was found primarily in rural counties in southern Florida (eg, Collier, Glades, Okeechobee), some urban counties along the eastern coast (eg, Martin, Flagler, Brevard), and some parts of the westernmost Panhandle (most notably, Escambia) (Figure 2, panel C). The 6 counties with the highest prevalence of elevated blood pressure were in southern Florida and included both interior agricultural counties (Highlands, Okeechobee, Collier) and urban coastal retirement-destination counties (St. Lucie, Indian River, Martin).

County	Diagnosed Hypertension, %	All Hypertension, Diagnosed and Undiagnosed, %	Elevated Blood Pressure With No Hypertension Diagnosis, %
Alachua	22.5	30.3	10.2
Baker	31.2	37.6	10.3
Bay	18.9	32.8	19.6
Bradford	32.1	41.9	12.9
Brevard	13.0	25.1	21.9
Broward	12.9	26.7	17.6
Calhoun	41.3	48.8	9.0
Charlotte	19.0	28.0	16.9
Citrus	18.8	36.1	22.8
Clay	20.0	27.8	14.0
Collier	3.7	13.4	27.2
Columbia	25.9	37.3	16.0
DeSoto	10.1	18.5	18.8
Dixie	36.7	47.7	13.2
Duval	30.6	36.2	9.7
Escambia	19.0	35.0	21.1
Flagler	15.9	28.6	21.5
Franklin	43.1	50.8	10.7
Gadsden	46.0	54.2	10.9
Gilchrist	26.9	38.1	14.6
Glades	12.9	22.4	22.4
Gulf	25.4	37.8	17.9
Hamilton	29.6	41.9	17.2
Hardee	9.4	20.0	20.8
Hendry	10.9	24.9	22.5
Hernando	16.8	27.1	17.3
Highlands	12.9	24.6	24.1
Hillsborough	18.6	23.1	9.6
Holmes	27.6	39.3	17.5
Indian River	7.8	19.1	25.4
Jackson	40.2	48.8	11.7
Jefferson	44.8	52.0	8.8
Lafayette	26.2	37.3	15.5
Lake	15.5	27.7	20.2
Lee	18.1	26.1	15.0
Leon	32.5	42.5	14.1
Levy	28.8	40.5	15.2
Liberty	42.5	50.7	10.5
Madison	40.9	49.7	12.1
Manatee	9.5	19.3	20.2
Marion	18.7	32.7	18.8
Martin	6.9	19.0	26.2
Miami-Dade	13.5	25.2	15.2
Monroe	11.4	26.6	21.0
Nassau	30.9	38.4	11.3
Okaloosa	16.7	29.5	19.1
Okeechobee	10.9	20.7	25.4
Orange	18.7	25.5	14.2
Osceola	14.6	23.2	15.2
Palm Beach	11.0	23.8	19.1
Pasco	19.5	25.0	9.8
Pinellas	22.6	28.8	7.5
Polk	14.9	24.7	16.9
Putnam	26.5	39.9	17.9
Santa Rosa	13.2	28.4	20.3
Sarasota	11.3	23.6	19.6
Seminole	15.5	23.1	14.6
St. Johns	15.0	23.4	14.9
St. Lucie	7.6	18.9	25.4
Sumter	18.3	35.1	22.5
Suwannee	25.4	38.0	17.8
Taylor	41.8	51.3	12.7
Union	26.1	36.9	14.6
Volusia	13.1	23.1	18.7
Wakulla	35.9	44.0	10.7
Walton	20.5	32.9	17.5
Washington	27.0	39.0	16.9

Among patients with hypertension, most were women (53.7%), white (65.6%), and non-Hispanic (76.7%). Among those with data on BMI (90.2% of hypertensive patients), 48.4% had a BMI of 30.0 or more, and 31.3% had a BMI of 25.0 to less than 30.0. The most prevalent comorbidities were dyslipidemia (38.9%), diabetes (24.2%), and coronary artery disease (13.3%); the prevalence of comorbidities differed by age group (Table 1).

Blood pressure data were available for 722,623 (86.2%) hypertensive patients. Mean (standard deviation [SD]) blood pressure in the population was 139 (19) mm Hg systolic and 80 (12) mm Hg diastolic. Increasing age was associated with increased mean systolic blood pressure and decreased mean diastolic blood pressure. Overall, 61.0% had uncontrolled blood pressure. Among hypertensive patients, the prevalence of blood pressure control was lowest among those aged 18 to 44 (28% controlled) but was generally similar (~40% to 42%) across older age groups.

**Figure 3:**
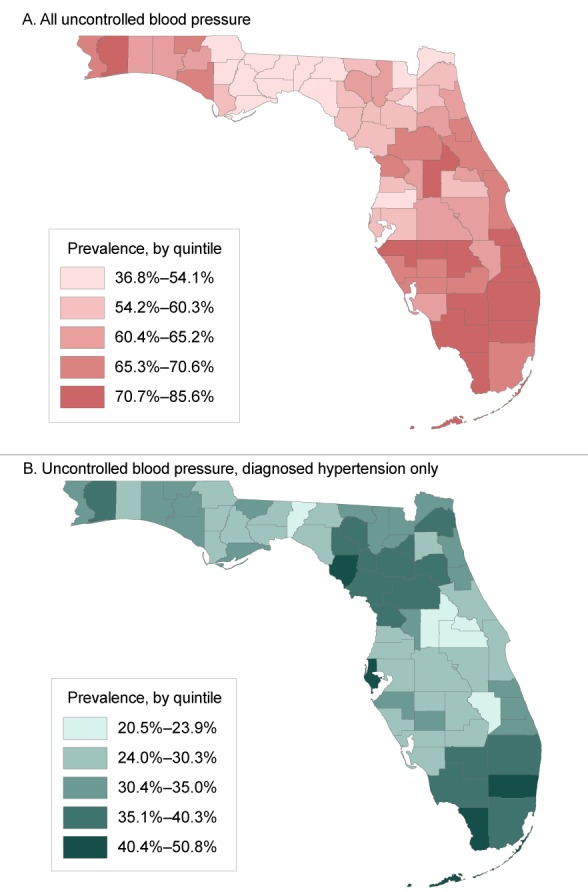
Prevalence, by quintile, of uncontrolled blood pressure (≥140/90 mm Hg) among hypertensive patients with at least 1 ambulatory visit or outpatient encounter recorded from January 1, 2012, through June 30, 2016, in OneFlorida, a partnership of 11 health systems and affiliated practices in Florida, by county. Panel A, prevalence of uncontrolled blood pressure, regardless of hypertension diagnosis; panel B, prevalence of uncontrolled blood pressure only among those with a hypertension diagnosis. **Table:** 

Collier County, in southwestern Florida, had the highest prevalence of uncontrolled blood pressure (85.6%), regardless of diagnosis, and the coastal-retirement destination counties of Monroe, Indian River, St. Lucie, and Martin each had a prevalence of 78% or more (Figure 3, panel A). Among patients with a hypertension diagnosis, we found the highest prevalence (50.8%) in Monroe County, followed by Dixie and Broward counties, each with a prevalence of 43% (Figure 3, panel B).

County	All Uncontrolled Blood Pressure, %	Uncontrolled Blood Pressure, Diagnosed Hypertension Only, %
Alachua	55.5	35.7
Baker	54.1	33.8
Bay	65.8	31.2
Bradford	53.7	35.7
Brevard	70.6	28.2
Broward	72.8	42.5
Calhoun	44.2	30.3
Charlotte	65.2	27.8
Citrus	70.2	38.2
Clay	59.3	28.7
Collier	85.6	38.6
Columbia	60.5	34.9
DeSoto	68.0	34.5
Dixie	57.5	42.5
Duval	54.2	35.4
Escambia	68.1	33.5
Flagler	68.4	32.6
Franklin	46.8	30.9
Gadsden	47.1	31.2
Gilchrist	60.1	39.0
Glades	67.0	27.0
Gulf	56.9	25.6
Hamilton	60.2	34.6
Hardee	72.2	30.0
Hendry	77.0	39.1
Hernando	59.1	25.9
Highlands	71.2	26.9
Hillsborough	55.8	24.9
Holmes	65.2	33.3
Indian River	79.7	31.7
Jackson	46.6	27.7
Jefferson	36.8	23.9
Lafayette	60.3	36.2
Lake	73.3	20.5
Lee	64.1	30.0
Leon	51.2	27.3
Levy	58.8	38.4
Liberty	43.3	27.1
Madison	43.2	25.2
Manatee	71.4	33.0
Marion	66.4	36.5
Martin	78.2	33.3
Miami-Dade	69.1	40.3
Monroe	80.7	50.8
Nassau	52.5	33.2
Okaloosa	65.0	29.9
Okeechobee	65.2	20.7
Orange	59.3	20.5
Osceola	62.6	25.8
Palm Beach	73.0	39.8
Pasco	53.0	26.8
Pinellas	54.4	41.3
Polk	64.8	28.3
Putnam	62.6	37.7
Santa Rosa	73.4	37.1
Sarasota	70.1	30.2
Seminole	61.1	21.8
St. Johns	63.3	32.7
St. Lucie	78.5	35.0
Sumter	63.9	34.2
Suwannee	62.2	35.9
Taylor	45.8	28.7
Union	56.6	33.4
Volusia	69.3	29.4
Wakulla	45.2	26.8
Walton	62.4	32.3
Washington	61.3	32.8

Data on prescribed medications were available for 519,879 patients, or 62.0% of the overall hypertensive population. Of these, 13.2% were prescribed no medications, 27.9% were prescribed medications other than antihypertensive medications, and 58.9% were prescribed at least 1 antihypertensive medication. Among those with diagnosed hypertension (Table 2), the most commonly prescribed medications were angiotensin-converting enzyme (ACE) inhibitors (39.6% of patients), β-blockers (34.5%), calcium channel blockers (29.7%), and thiazide diuretics (29.7%). Among those with elevated blood pressure but no hypertension diagnosis, β-blockers (13.1%), ACE inhibitors (9.7%), and calcium channel blockers (9.5%) were the most commonly prescribed antihypertensive medications (Table 3).

**Table 2:** Antihypertensive Medications Prescribed Among Patients With ICD-9-CM–Diagnosed or ICD-10-CM–Diagnosed Hypertension in OneFlorida, January 2012–June 2016^a^

Medication Class	Age Group, y				Total
	18–44	45–64	65–74	≥75	
**Calcium channel blockers**	10,840 (22.7)	47,288 (28.7)	24,476 (31.9)	20,863 (35.8)	103,467 (29.7)
**Angiotensin-converting enzyme (ACE) inhibitors**	17,244 (36.0)	71,598 (43.4)	29,560 (38.5)	19,309 (33.1)	137,711 (39.6)
**Angiotensin receptor blockers (ARBs)**	4,538 (9.5)	27,005 (16.4)	16,138 (21.0)	12,976 (22.2)	60,657 (17.4)
**β-Blockers**	11,980 (25.0)	52,366 (31.7)	29,650 (38.6)	25,986 (44.5)	119,982 (34.5)
**Diuretics**					
Thiazides	12,970 (27.1)	53,072 (32.2)	22,928 (29.9)	14,322 (24.5)	103,292 (29.7)
Aldosterone antagonists	980 (2.0)	4,705 (2.9)	2,612 (3.4)	1,978 (3.4)	10,275 (3.0)
Other	3,324 (6.9)	18,102 (11.0)	12,053 (15.7)	12,938 (22.2)	46,417 (13.3)
**Others**	3,245 (6.8)	11,599 (7.0)	5,231 (6.8)	4,481 (7.7)	24,556 (7.1)

Abbreviations: ICD-9-CM, *International Classification of Diseases, Ninth Revision, Clinical Modification* (11); ICD-10-CM, *International Classification of Diseases, Tenth Revision, Clinical Modification* (12).

a Values are number (percentage) among 347,948 patients with diagnosed hypertension for whom data on prescriptions were available.

**Table 3:** Antihypertensive Medications Prescribed Among Patients With an Elevated Blood Pressure But No ICD-9-CM–Diagnosed or ICD-10-CM–Diagnosed Hypertension in OneFlorida, January 2012–June 2016^a^

Medication Class	Age Group, y				Total
	18–44	45–64	65–74	≥75	
**Calcium channel blockers**	1,230 (2.8)	5,353 (8.0)	4,043 (12.8)	5,625 (19.0)	16,251 (9.5)
**Angiotensin-converting enzyme (ACE) inhibitors**	1,367 (3.1)	6,851 (10.3)	4,160 (13.2)	4,371 (14.7)	16,749 (9.7)
**Angiotensin receptor blockers (ARBs)**	476 (1.1)	3,935 (5.9)	3,523 (11.2)	4,661 (15.7)	12,595 (7.3)
**β-Blockers**	2,174 (4.9)	7,339 (11.0)	5,531 (17.5)	7,505 (25.3)	22,549 (13.1)
**Diuretics**					
Thiazides	898 (2.0)	4,501 (6.8)	3,063 (9.7)	3,245 (10.9)	11,707 (6.8)
Aldosterone antagonists	193 (0.4)	506 (0.8)	315 (1.0)	386 (1.3)	1,400 (0.8)
Other	541 (1.2)	2,252 (3.4)	1,756 (5.6)	2,975 (10.0)	7,524 (4.4)
**Others**	676 (1.5)	1,371 (2.1)	838 (2.7)	1,188 (4.0)	4,073 (2.4)

Abbreviations: ICD-9-CM, *International Classification of Diseases, Ninth Revision, Clinical Modification* (11); ICD-10-CM, *International Classification of Diseases, Tenth Revision, Clinical Modification* (12).

a Values are as number (percentage) among 171,931 undiagnosed patients with medication data.

## Discussion

Our study, to our knowledge, is the largest cross-sectional study of Floridians with hypertension and among the first wide-scale efforts to use clinical data from real-world usual care in a health system that spans an entire state. We observed an overall hypertension prevalence of 27.9% among adult patients with at least 1 ambulatory visit or outpatient encounter. Of these, approximately two-thirds had diagnosed hypertension, and the remainder had elevated blood pressure but no diagnosis. This overall prevalence is lower than the 33.5% of adults in the 2015 BRFSS who reported having ever been told they had high blood pressure (13). As we anticipated, prevalence differed by age group, with the lowest prevalence among those aged 18 to 44 and a higher prevalence among older patients. The proportion with elevated blood pressure but no hypertension diagnosis (among the overall hypertensive cohort) decreased from 49.6% among those aged 18 to 44 years to 27.0% among those aged 75 or older, suggesting that older patients were more likely to receive a diagnosis. We also observed a higher overall hypertension prevalence among women (53.7%) than among men (46.3%); in contrast, BRFSS estimates show a higher prevalence of hypertension among men in Florida (13). The differences between our results and those of BRFSS may reflect differences between the 2 cohorts in the distribution of older women; hypertension prevalence and average blood pressure are higher among men than women younger than 45, whereas the reverse is true among those aged 65 or older (4,14–16). Alternatively, the higher prevalence among women may reflect their more frequent interaction with the health care system, resulting in a greater opportunity for diagnosing hypertension. Finally, among all patients with hypertension in our study, 21.0% were black and 65.6% were white. However, the black population was overrepresented in the younger age groups and underrepresented in the older age groups. These racial distributions are generally consistent with population distributions in Florida, where the black population is approximately 15% of the overall adult population but only 9% of those aged 65 to 74 and less than 7% of those aged 75 or older (17). The reason for this racial distribution is not clear but may relate to racial disparities in migration patterns into and out of the state. Alternatively, these data may reflect the well-known disparities in hypertension-related mortality between black and white Americans (18).

Our data source allowed for mapping of hypertension prevalence. We observed the highest overall hypertension prevalence in northern Florida, an area that is demographically and geographically similar to much of the southeastern United States, which has among the highest rates of hypertension in the nation. Not coincidentally, the areas in northern Florida also comprise the southern border of the US stroke belt, which historically has had higher levels of age-adjusted stroke mortality than other areas of the United States. Our results are also consistent with those of previous studies that combined NHANES and BRFSS data and showed that the highest prevalence of hypertension in Florida is in the northern part of the state (19). Likewise, recent data from the Reasons for Geographic and Racial Differences in Stroke project show that the highest hypertension prevalence among both blacks and whites in Florida is in the northwestern part of the state (20). Patients with elevated blood pressure but no hypertension diagnosis were concentrated in multiple areas, including rural areas of southern Florida, eastern coastal counties, and some rural counties of the western Panhandle. BRFSS generates county-level estimates of hypertension prevalence based on patient self-report, but it does not capture data on hypertension among those with no diagnosis or knowledge of having the disease. Thus, our approach is useful for pinpointing regions that need programs to reduce the prevalence of uncontrolled hypertension and other chronic diseases that contribute to the large and growing racial/ethnic disparities in diseases (eg, stroke [21]), and in life expectancy (22,23).

Finally, we explored data on common antihypertensive medications to better understand their real-world use among Floridians with hypertension. Not surprisingly, the 4 most commonly used classes of medication were ACE inhibitors, β-blockers, calcium channel blockers, and diuretics (especially thiazides). These data appear consistent with national estimates based on NHANES data from 2001 to 2010 (14). Our data could be useful, for example, in identifying and targeting disparities in medication selection or dosing among various segments of the population.

Our study and data source have several strengths. First, we used clinical data, rather than patient-reported data, to identify hypertension cases. Our approach allowed for ascertaining cases of physician-diagnosed hypertension and cases of hypertension with no documented diagnosis. Detection of hypertension, particularly cases for which no diagnosis is documented, may allow for more focused interventions in the health care system, public health sector, or both, to prevent the adverse sequelae of uncontrolled blood pressure. Second, the detailed information on patients in the OneFlorida Data Trust allowed for a more granular view of hypertension in Florida than provided by surveys such as BRFSS and NHANES. Our study produced data on overall hypertension prevalence; prevalence by age, sex, race/ethnicity, and geographic region; and detailed characterization by age group. Areas of future study include analyzing time trends in hypertension prevalence, blood pressure control, antihypertensive treatment, and other parameters related to hypertension for the overall population in Florida and subgroups. Furthermore, development and validation of outcome measures in the PCORnet common data model will allow for a transition from descriptive studies of individual populations (eg, Florida) to use of inferential statistics to better understand care and treatment effects across larger populations from which these patients are sampled. Third, because these data are collected as part of routine care, they do not require substantial financial investment to update regularly, beyond the initial and ongoing infrastructure costs for the clinical data research network. Although such data are not expected to supplant ongoing surveillance efforts, they may be a useful and cost-effective adjunct to identifying disparities and areas of need for public health investment.

Several limitations should also be noted. First, our data are not necessarily generalizable to the entire adult population of Florida or adult populations in other states. Although OneFlorida partners provide care for nearly half of all Floridians, outpatient data were available only for approximately 25% of Floridian adults when we conducted this study. Moreover, systematic differences exist between persons who engage the health care system and those who do not. For example, patients in the health care system are sicker than the general population, which could prompt overestimation of hypertension prevalence in the general population. Conversely, some adults with low socioeconomic status and who lack medical insurance (eg, low-income, childless adults) may forgo routine outpatient visits. Data on such patients, who are at elevated risk of having both hypertension and uncontrolled blood pressure (24), would not be captured in OneFlorida, thus resulting in underestimation of prevalence. Nevertheless, to our knowledge, OneFlorida is the largest clinical data repository for adults receiving health care in Florida. Second, we excluded potential hypertension cases in which care was received only in an inpatient or emergency department setting. Third, our definition of hypertension included a blood pressure of 140/90 mm Hg or more. This definition is consistent with definitions in other large representative data sets (ie, NHANES); however, we cannot exclude the possibility of generating false-positive cases of hypertension by using this definition, either because blood pressure was improperly measured or inaccurately recorded or because of some other patient- or visit-specific factor (eg, patient sought care for pain) that may have accounted for a lone elevated blood pressure. Thus, our definition of hypertension, which included patients with no hypertension diagnosis, may have resulted in the overestimation of undiagnosed hypertension. Finally, general limitations exist for using EHR data, particularly diagnostic codes, in research, because such data are collected primarily for the purposes of billing. For example, use of ICD codes for identifying hypertension cases have reasonably high but imperfect sensitivity and specificity (25,26).

We analyzed data on more than 3 million patients in OneFlorida for evidence of hypertension and found an overall prevalence of 27.9%. We further provided proof-of-concept for the use of a large, integrated, clinical data repository in augmenting surveillance of chronic diseases such as hypertension. The advantages to our approach include the use of existing clinical data (representing usual care), which allows for identifying patients with uncontrolled blood pressure but no hypertension diagnosis, and the ability to parse outcomes on the basis of detailed patient-level data such as age, race/ethnicity, and geographic location. As the nation moves toward the goal of creating health systems in which patient care informs evidence as much as evidence informs patient care, pragmatic research networks, like OneFlorida, may be increasingly relied on to inform future health care, support the design and conduct of clinical trials, and enhance public health efforts.

## References

[1] Kearney PM , Whelton M , Reynolds K , Muntner P , Whelton PK , He J . Global burden of hypertension: analysis of worldwide data. Lancet 2005;365(9455):217–23. 10.1016/S0140-6736(05)70151-3 15652604

[2] Mills KT , Bundy JD , Kelly TN , Reed JE , Kearney PM , Reynolds K , Global disparities of hypertension prevalence and control: a systematic analysis of population-based studies from 90 countries. Circulation 2016;134(6):441–50. 10.1161/CIRCULATIONAHA.115.018912 27502908 PMC4979614

[3] Patel SA , Winkel M , Ali MK , Narayan KM , Mehta NK . Cardiovascular mortality associated with 5 leading risk factors: national and state preventable fractions estimated from survey data. Ann Intern Med 2015;163(4):245–53. 10.7326/M14-1753 26121190

[4] Benjamin EJ , Blaha MJ , Chiuve SE , Cushman M , Das SR , Deo R , Heart disease and stroke statistics — 2017 update: a report from the American Heart Association. Circulation 2017;135(10):e146–603. 10.1161/CIR.0000000000000485 28122885 PMC5408160

[5] Florida Department of Health. Florida state health improvement plan, 2012–2015. 2012. http://www.floridahealth.gov/about-the-department-of-health/_documents/state-health-improvement-plan.pdf. Accessed June 5, 2017.

[6] Sampson UK , Kaplan RM , Cooper RS , Diez Roux AV , Marks JS , Engelgau MM , Reducing health inequities in the U.S.: recommendations from the NHLBI’s Health Inequities Think Tank Meeting. J Am Coll Cardiol 2016;68(5):517–24. 10.1016/j.jacc.2016.04.059 27470459 PMC4968582

[7] Shenkman E , Hurt M , Hogan W , Carrasquillo O , Smith S , Brickman A , OneFlorida Clinical Research Consortium: linking a clinical and translational science institute with a community-based distributive medical education model. Acad Med 2017. 10.1097/ACM.0000000000002029 29045273 PMC5839715

[8] Fleurence RL , Curtis LH , Califf RM , Platt R , Selby JV , Brown JS . Launching PCORnet, a national patient-centered clinical research network. J Am Med Inform Assoc 2014;21(4):578–82. 10.1136/amiajnl-2014-002747 24821743 PMC4078292

[9] OneFlorida Clinical Research Consortium. About the consortium. 2013. http://onefloridaconsortium.org/about-the-consortium/. Accessed April 8, 2017.

[10] Yuan J , Malin B , Modave F , Guo Y , Hogan WR , Shenkman E , Towards a privacy preserving cohort discovery framework for clinical research networks. J Biomed Inform 2017;66:42–51. 10.1016/j.jbi.2016.12.008 28007583 PMC5316314

[11] National Center for Health Statistics, Centers for Disease Control and Prevention. International classification of diseases, ninth revision, clinical modification. http://www.cdc.gov/nchs/icd/icd9cm.htm. Accessed January 8, 2018.

[12] National Center for Health Statistics, Centers for Disease Control and Prevention. International classification of diseases, tenth revision, clinical modification. https://www.cdc.gov/nchs/icd/icd10cm.htm. Accessed January 8, 2018.

[13] Florida Department of Health. Florida Behavioral Risk Factor Surveillance System (BRFSS) 2015 data book. 2016. http://www.floridahealth.gov/statistics-and-data/survey-data/behavioral-risk-factor-surveillance-system/reports/_documents/2015-brfss.pdf. Accessed June 5, 2017.

[14] Gu Q , Burt VL , Dillon CF , Yoon S . Trends in antihypertensive medication use and blood pressure control among United States adults with hypertension: the National Health And Nutrition Examination Survey, 2001 to 2010. Circulation 2012;126(17):2105–14. 10.1161/CIRCULATIONAHA.112.096156 23091084

[15] Gu Q , Burt VL , Paulose-Ram R , Dillon CF . Gender differences in hypertension treatment, drug utilization patterns, and blood pressure control among US adults with hypertension: data from the National Health and Nutrition Examination Survey 1999–2004. Am J Hypertens 2008;21(7):789–98. 10.1038/ajh.2008.185 18451806

[16] McDonald M , Hertz RP , Unger AN , Lustik MB . Prevalence, awareness, and management of hypertension, dyslipidemia, and diabetes among United States adults aged 65 and older. J Gerontol A Biol Sci Med Sci 2009;64(2):256–63. 10.1093/gerona/gln016 19181717 PMC2655011

[17] Leng B , Jin Y , Li G , Chen L , Jin N . Socioeconomic status and hypertension: a meta-analysis. J Hypertens 2015;33(2):221–9. 10.1097/HJH.0000000000000428 25479029

[18] Kung HC , Xu J . Hypertension-related mortality in the United States, 2000–2013. NCHS Data Brief 2015;(193):1–8. 25932893

[19] Olives C , Myerson R , Mokdad AH , Murray CJ , Lim SS . Prevalence, awareness, treatment, and control of hypertension in United States counties, 2001–2009. PLoS One 2013;8(4):e60308. 10.1371/journal.pone.0060308 23577099 PMC3618269

[20] Loop MS , Howard G , de Los Campos G , Al-Hamdan MZ , Safford MM , Levitan EB , Heat maps of hypertension, diabetes mellitus, and smoking in the continental United States. Circ Cardiovasc Qual Outcomes 2017;10(1):e003350. 10.1161/CIRCOUTCOMES.116.003350 28073852 PMC5234692

[21] Sacco RL , Gardener H , Wang K , Dong C , Ciliberti-Vargas MA , Gutierrez CM , Racial-ethnic disparities in acute stroke care in the Florida-Puerto Rico Collaboration to Reduce Stroke Disparities Study. J Am Heart Assoc 2017;6(2):e004073. 10.1161/JAHA.116.004073 28196814 PMC5523741

[22] Dwyer-Lindgren L , Bertozzi-Villa A , Stubbs RW , Morozoff C , Kutz MJ , Huynh C , US county-level trends in mortality rates for major causes of death, 1980–2014. JAMA 2016;316(22):2385–401. 10.1001/jama.2016.13645 27959996 PMC5576343

[23] Dwyer-Lindgren L , Bertozzi-Villa A , Stubbs RW , Morozoff C , Mackenbach JP , van Lenthe FJ , Inequalities in life expectancy among US counties, 1980 to 2014: temporal trends and key drivers. JAMA Intern Med 2017;177(7):1003–11. 10.1001/jamainternmed.2017.0918 28492829 PMC5543324

[24] Florida Office of Economic and Demographic Research. Population and demographic data by county, age, race, sex and Hispanic origin. 2017. http://edr.state.fl.us/Content/population-demographics/data/Medium_Projections_ARSH.pdf. Accessed August 15, 2017.

[25] Bullano MF , Kamat S , Willey VJ , Barlas S , Watson DJ , Brenneman SK . Agreement between administrative claims and the medical record in identifying patients with a diagnosis of hypertension. Med Care 2006;44(5):486–90. 10.1097/01.mlr.0000207482.02503.55 16641668

[26] Birman-Deych E , Waterman AD , Yan Y , Nilasena DS , Radford MJ , Gage BF . Accuracy of ICD-9-CM codes for identifying cardiovascular and stroke risk factors. Med Care 2005;43(5):480–5. 10.1097/01.mlr.0000160417.39497.a9 15838413

